# Eau Brocchieri

**Published:** 1846-03-01

**Authors:** 


*Eau Brocchieri.*—It would be an interminable, as well as profitless task to undertake to expose all the contrivances with which charlatanry succeeds in practicing successfully upon the inexhaustible capabilities of human credulity. Generally we shall abstain from any interference with popular nostrums. We have no hopes of improvement until the appetite for the marvellous and ridiculous has been indulged to satiety. We are induced to deviate from general rule in reference to the wonderful styptic of which so much is said in the newspapers, because it appears to be a common impression in, as well as out of the profession, that it has emanated from a scientific member of the profession, and that its virtue has been properly attested by satisfactory experiments before competent observers. The whole matter, however, is a gross humbug and imposition. We have examined the article by the senses. It appears to be water slightly impregnated with tar! It has no pungent, astringent nor active properties whatever. The following extract from a clinical lecture by Dr. Mott, of New-York, which we quote from the New-York Medical and Surgical Reporter, gives the opinion of one fully competent to ascertain what efficacy it possesses:

"I knew M. Brocchieri when I was in Paris; he is an uneducated man, and a perfect charlatan. When his discovery was made known in Paris, it created some stir;—and I made several experiments with it, in connection with several other gentlemen, one of whom was engaged in the preparation of the water. The subjects of the experiments, were strong and healthy sheep, upon whose carotid arteries we operated, and we found that its power to stop hæmorrhage was next to nothing, and where the bleeding was arrested, it was principally from the pressure made by the large quantities of lint, with which the wound was filled. Therefore, I say, as the result of my experience, that the styptic powers of this preparation are not to be relied upon, for a moment: that it is infinitely less useful than an infusion of rhatany, or tannin, and that it can never take the place of needles and ligatures.

The other qualities that have been ascribed to it, of curing disease, and arresting hæmoptysis, are equally non-existant.

Doctor M. having occasion to remove a tumor from the cheek of a female, during his lecture, applied some of the nostrum, but without the least effect."

